# No association of genetic polymorphisms in *CYP1B1* with primary open-angle glaucoma: a meta- and gene-based analysis

**Published:** 2012-03-31

**Authors:** Shuqian Dong, Jingyun Yang, Weihong Yu, Pravina Kota, Xiaobo Xia, Huizhuo Xu

**Affiliations:** 1 Department of Ophthalmology, Xiangya Hospital, Central South University, Changsha, Hunan, China; 2 Methodology Center, Pennsylvania State University, State College, PA; 3 Department of Ophthalmology, Peking Union Medical College Hospital, Chinese Academy of Medical Science, Beijing, China; 4 Department of Biostatistics and Epidemiology, University of Oklahoma Health Sciences Center, Oklahoma City, OK

## Abstract

**Purpose:**

To examine the effects of genetic polymorphisms in cytochrome P450, subfamily 1, polypeptide 1 (*C1P1B1*) on primary open-angle glaucoma (POAG).

**Methods:**

A systematic literature search was performed, and random-effects meta-analyses were used to evaluate genetic polymorphisms in *CYP1B1* with POAG. A gene-based analysis was conducted to investigate the cumulative effects of genetic polymorphisms in *CYP1B1*.

**Results:**

A total of six studies from published papers were included in our analysis. Random-effects meta-analyses failed to detect any significant association of POAG with genetic polymorphisms in *CYP1B1*, including rs180040, rs1056836, rs10012, rs1056827, rs1056837, and rs2567206. The gene-based analysis indicated that the cumulative effect of genetic polymorphisms in *CYP1B1* is not associated with POAG (p>0.50).

**Conclusions:**

We did not find any evidence of strong association of POAG with *CYP1B1* genetic polymorphisms and their cumulative effect.

## Introduction

Glaucoma is defined as a group of heterogeneous, complex disorders characterized by a progressive loss of retinal ganglion cells; it is a major cause of irreversible blindness [1,2]. Primary open-angle glaucoma (POAG) is the most common form of glaucoma, leading to an estimated 3.3 million cases of bilateral blindness worldwide [3]. POAG is defined by an open, normal appearing anterior chamber angle and raised intraocular pressure (IOP), with no other underlying disease. The onset of the disease is not obvious to the patient until there is appreciable and irreversible loss of the field of vision.

POAG is a complex disease caused by multiple genetic and environmental factors, as well as their interactions [4-8]. A previous study estimated that 72% of all POAG cases exhibited an inherited or familial form of the disease, which does not show a clear pattern of Mendelian inheritance [9]. Mutations, polymorphisms, and copy number variations (CNVs) could contribute to the pathogenesis of POAG. To date, more than 20 genetic loci have been implicated in its development [10]. Linkage analysis has identified two POAG-causing genes, myocilin (*MYOC*) [5] and optineurin (*OPTN*) [7]. More than 70 *MYOC* mutations have been reported to contribute to the pathogenesis of POAG [10], and *OPTN* mutations have been associated with normal tension glaucoma (NTG) [7]. Variants in these two genes account for about 5% of POAG in the population [7,11]. Previous studies also have reported the association of POAG with mutations in WD repeat domain 36 (*WDR36*) [12] and neurotrophin-4 (*NTF4*) [13,14]; however, their roles in the pathogenesis of POAG is controversial [15]. CNVs are defined as insertions or deletions of large segments of DNA, from 1 kb up to several Mb; they have been found to contribute to many complex disorders, such as autism [16,17], schizophrenia [18], and Crohn’s disease [19]. A recent study identified 11 validated CNVs in patients with POAG, but not in age-matched controls, suggesting the potential role that the CNV-implemented genes might play in the pathogenesis of POAG.

Mutations in *CYP1B1* (cytochrome P450, family 1, subfamily B, polypeptide 1) have also been identified in POAG patients and may be suggested as a modifier of POAG in carriers of *MYOC* mutations [20,21]. Most studies on the effect of *CYP1B1* on POAG have investigated only genetic mutations in this gene [20,22-29]. Although a few studies have evaluated the association of single-nucleotide polymorphisms (SNPs) of *CYP1B1* with PAOG, the results are conflicting [22,30-34].

In this study, we conducted a systematic literature search of published studies examining the association of genetic polymorphisms of *CYP1B1* with POAG and conducted meta-analyses of SNPs in this gene. POAG is a complex disorder, and it is highly likely that individual SNPs may contribute little to its onset and development. However, their cumulative effects may be significant. Therefore, we conducted a gene-based analysis to investigate the cumulative effects of the genetic polymorphisms in *CYP1B1* on POAG.

## Methods

### Search strategy and study selection

In October 2011, we conducted an extensive literature search of MEDLINE, Cochrane Library, Web of Science, and Google Scholar. Search terms included “primary open angle glaucoma,” “*CYP1B1,*” “SNP,” “polymorphism,” and “POAG.” The following inclusion criteria were used in the search: 1) studies on human subjects; 2) studies on POAG; 3) reported association (or have data available to calculate the association) of genetic polymorphism of individual SNPs in *CYP1B1* with POAG; and 4) provided odds ratios and their variance (or data to calculate the variance) or genotype frequency among participants with and without POAG. All potentially relevant publications were retrieved and evaluated for inclusion. We also hand-searched references of all relevant publications for additional studies missed by the database search. Our search was restricted to studies published in the English language. Two authors (S.D. and J.Y.) performed the search independently. Disagreement over eligibility of a study was resolved by the evaluation of a third reviewer (W.Y.) and discussion until a consensus was reached.

### Statistical analysis

We used odds ratio (OR) as a measure of the association of the genetic polymorphisms in *CYP1B1* with POAG. ORs were used as provided in the papers or were calculated from genotype frequency data, and they were logarithmically transformed to improve normality. Standard errors were derived from the confidence intervals (CI) reported in each study. Random-effects models were used to calculate ORs and their corresponding 95% CIs. The z-test was used to calculate the p-value of the overall effect, and a forest plot was used to present the calculated pooled ORs and their 95% CIs. In the forest plot, each study was represented by a square whose area was proportional to the weight of the study. The weight of each study was determined by taking the inverse of the variance of each study. The overall effect from meta-analysis is represented by a diamond in the forest plot; the width of the diamond represents the 95% CI for the estimated OR. We used Q statistics to assess between-study heterogeneity. Because Q statistics are underpowered, we considered studies to be homogeneous if p>0.1. Publication bias was assessed visually using a funnel plot and tested with Egger’s regression test.

To assess the overall association of *CYP1B1* with POAG, we conducted a gene-based analysis, using the reported p-values of the association of genetic polymorphisms in *CYP1B1* with POAG and the p-values from our meta-analysis. This association was assessed using four popular p-value combination methods: Fisher’s method, the Simes method, the modified inverse normal method, and the truncated product method (TPM). A detailed description of the four methods has been published elsewhere [35]. We conducted 100,000 simulations to estimate the p-value using TPM, because the p-values of the association of individual SNPs within *CYP1B1* with POAG are most likely to be dependent.

Meta-analysis was performed using Stata 11.2 (StataCorp LP, College Station, TX). All other analyses were performed using Matlab 7.10.0.499 (The MathWorks, Inc., Natick, MA).

## Results

### Literature search and eligible studies

**Figure 1:**
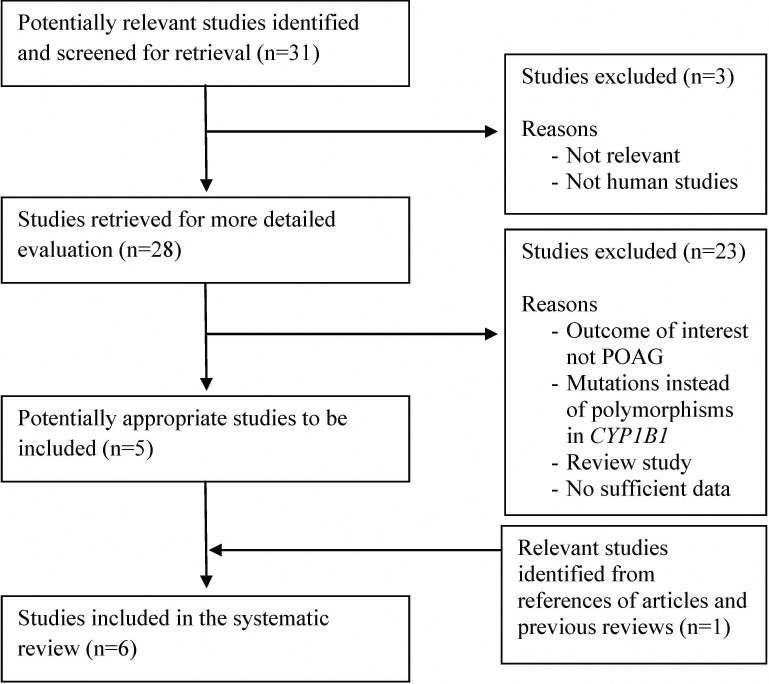
Flow diagram of studies included in the systematic review. Note: Please see the Methods section for additional details.

A flow diagram showing the selection process of studies included in our analysis is shown in Figure 1. Our initial search, using our pre-defined search strategy, identified a total of 31 potential studies. After reviewing each abstract, three studies were excluded, because they either were irrelevant or were not conducted on human subjects. The remaining 28 studies were retrieved for more detailed review. An additional 23 studies were excluded because the outcome of interest was not POAG, the study examined mutations instead of genetic polymorphisms, the study was a review, or there was insufficient data. A review of references for the remaining five studies identified one more relevant study. A total of six studies from published papers met the eligibility criteria and were included in our analyses [22,30-34].

All qualified studies were published after 2005, and they had sample sizes ranging from 271 to 1,758 participants. The reported prevalence of POAG ranged from 47% to 83% (Table 1). Of these six studies, five reported association results for rs180040 and rs1056836, three for rs10012, and two for rs1056827, rs1056837, and rs2567206. These studies were included in the corresponding meta-analysis for each of the six SNPs. The combined study population included 3,294 participants in the meta-analysis of rs180040 and rs1056836, 930 in rs10012, and 571 in rs1056827, rs1056837, and rs2567206. In addition to these six SNPs, the association between POAG and 14 other SNPs in *CYP1B1* was reported in single individual studies [22,30,32]. The results from individual studies and our meta-analysis were used to conduct our gene-based analysis.

**Table 1:** Basic characteristics of all studies.

**Study**	**Year of publication**	**Study population**	**Age**	**Prevalence of POAG**
[30]	2006	200 POAG patients and 100 controls in India	mean age 52.4±19.3	67%
[31]	2008	264 POAG patients and 95 controls in India	mean age 55.7±16.8	74%
[32]	2010	860 POAG patients and 898 controls in Australia	mean age 70.7	49%
[33]	2010	405 POAG patients and 201 controls in Hong Kong	mean age 57.8 and 69.8 for POAG patients and controls, respectively	67%
[34]	2005	224 POAG patients and 47 controls in France	-	83%
[22]	2010	339 POAG patients and 376 controls in Germany	mean age 66.9±13.4 and 73.9±6.4 for POAG patients and controls, respectively	47%

### Assessment of publication bias

**Figure 2:**
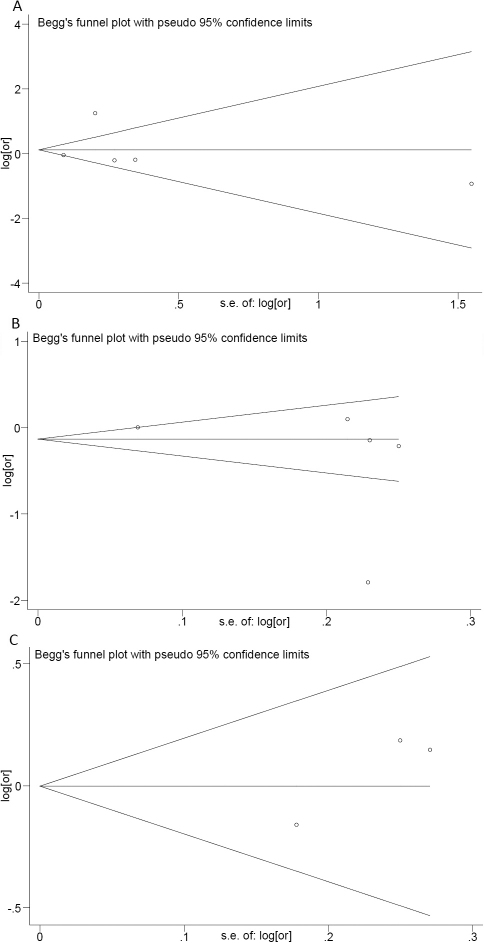
Begg’s funnel plots of random effects meta-analysis of the association of genetic polymorphisms in *CYP1B1* with primary open-angle glaucoma (POAG). The horizontal line in the figure represents the overall estimated log-transformed odds ratio. The two diagonal lines represent the pseudo 95% confidence limits of the effect estimate. **A**: Funnel plot for random effects meta-analysis of rs180040 with POAG. **B**: Funnel plot for random effects meta-analysis of rs1056836 with POAG. **C**: Funnel plot for random effects meta-analysis of rs10012 with POAG.

Funnel plots and Egger’s test were used to assess publication bias (Figure 2A-C). No publication bias was detected for the meta-analyses of rs180040 (t=0.22, 95% CI: −7.66–8.79; p=0.84), rs1856836 (t=-1.02, 95% CI: −13.26–6.83; p=0.38), or rs10012 (t=3.84, 95% CI: −8.89–16.60; p=0.16). Due to the limited number of studies, publication bias could not be assessed for the meta-analyses of rs1056827, rs1056837, and rs2567206.

### Association of individual SNPs with POAG

**Figure 3:**
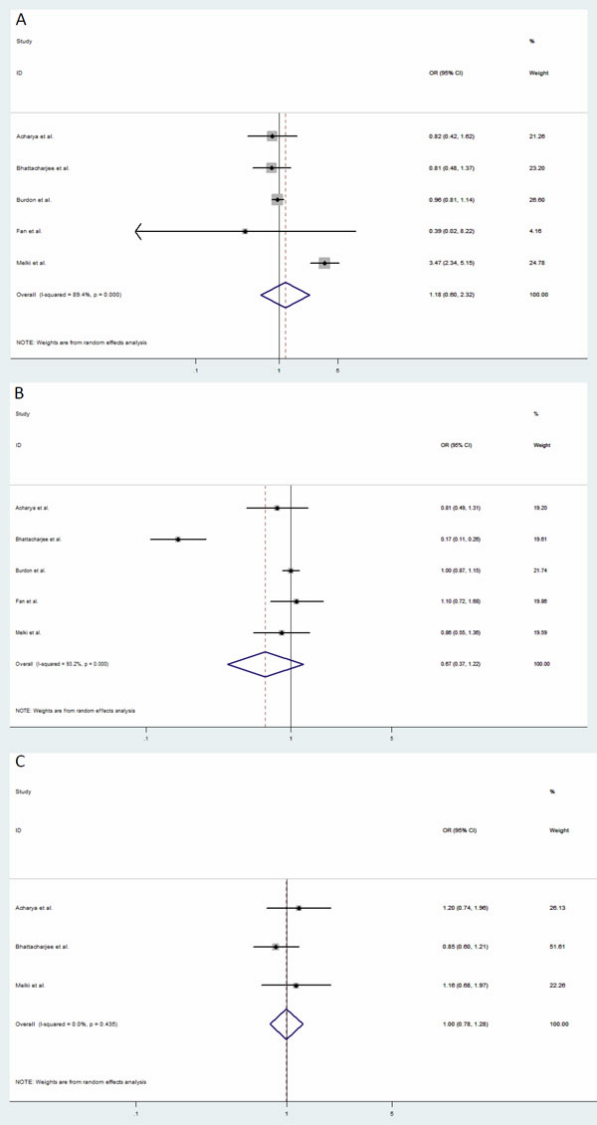
Forest plots of estimates of odds ratios of the association of genetic polymorphisms in *CYP1B1* with primary open-angle glaucoma (POAG). Each study was represented by a square whose area was proportional to the weight of the study. The overall effect from meta-analysis is represented by a diamond whose width represents the 95% CI for the estimated OR. **A**: Estimates of odds ratio of rs180040 in *CYP1B1* with POAG. **B**: Estimates of odds ratio of rs1056836 in *CYP1B1* with POAG. **C**: Estimates of odds ratio of rs10012 in *CYP1B1* with POAG.

Five studies provided results on the association of rs180040 with POAG. Random-effects meta-analysis provided an estimated odds ratio of 1.18 (95% CI: 0.60–2.32; p=0.63), indicating no significant association of the SNP with POAG (Figure 3A, Table 2A). There was significant between-study heterogeneity (Q=37.85, p<0.001).

**Table 2:** Meta-analysis of the association of SNPs with POAG.

**Study**	**Weights**	**OR (95% CI)**	**p-value**
**A. **rs180040			
[30]	21.26	0.82 (0.42–1.62)	0.57
[31]	23.20	0.81 (0.48–1.38)	0.43
[32]	26.60	0.96 (0.81–1.14)	0.62
[33]	4.16	0.39 (0.02–8.22)	0.55
[34]	24.78	3.48 (2.34–5.15)	5.89×10^-10^
Total	-	1.18 (0.60–2.32)	0.63
**B. **rs1056836			
[30]	19.20	0.81 (0.49–1.32)	0.39
[31]	19.61	0.17 (0.11–0.26)	4.31×10^-15^
[32]	21.74	1.00 (0.88–1.15)	0.98
[33]	19.86	1.10 (0.72–1.68)	0.66
[34]	19.59	0.86 (0.55–1.36)	0.53
Total	-	0.67 (0.37–1.22)	0.19
**C. **rs10012			
[30]	26.13	1.20 (0.74–1.97)	0.46
[31]	51.61	0.85 (0.60–1.21)	0.37
[34]	22.26	1.16 (0.68–1.97)	0.59
Total	-	1.00 (0.78–1.28)	0.99
**D. **rs1056827			
[30]	54.00	1.20 (0.74–1.97)	0.71
[34]	46.00	1.16 (0.68–1.97)	0.79
Total	-	1.18 (0.83–1.70)	0.36
**E. **rs1056837			
[30]	45.88	0.77 (0.47–1.26)	0.30
[34]	54.12	0.89 (0.57–1.40)	0.61
Total	-	0.83 (0.60–1.16)	0.28
**F. **rs2567206			
[30]	37.52	0.85 (0.50–1.46)	0.56
[34]	62.48	1.32 (0.94–1.83)	0.10
Total	-	1.12 (0.74–1.69)	0.60

Five studies provided results on the association of rs1056836 with POAG. Random-effects meta-analysis provided an estimated odds ratio of 0.67 (95% CI: 0.37–1.11; p=0.19), indicating no significant association with POAG (Figure 3B, Table 2B). There was significant between-study heterogeneity (Q=58.63, p<0.001).

Three studies provided results on the association of rs10012 with POAG. Random-effects meta-analysis provided an estimated odds ratio of 1.00 (95% CI: 0.78–1.28; p=0.99), indicating no significant association with POAG (Figure 3C, Table 2C). There was no between-study heterogeneity (Q=1.66, p=0.44).

Two studies provided results on the association of rs1056827 with POAG. Random-effects meta-analysis provided an estimated odds ratio of 1.18 (95% CI: 0.83–1.70; p=0.36), indicating no significant association with POAG (Table 2D). There was no between-study heterogeneity (Q=0.01, p=0.92).

Two studies provided results on the association of rs1056837 with POAG. Random-effects meta-analysis provided an estimated odds ratio of 0.83 (95% CI: 0.60–1.16; p=0.28), indicating no significant association with POAG (Table 2E). There was no between-study heterogeneity (Q=0.17, p=0.68).

Two studies provided results on the association of rs2567206 with POAG. Random-effects meta-analysis provided an estimated odds ratio of 1.12 (95% CI: 0.74–1.69; p=0.60), indicating no significant association with POAG (Table 2F). There was no between-study heterogeneity (Q=1.83, p=0.18).

In addition to these six SNPs, the association of POAG with 14 other SNPs in *C1P1B1* was reported in individual studies. The results from these studies, together with the meta-analysis results obtained from this study, are summarized in Table 3.

**Table 3:** Association of individual SNPs in *CYP1B1* with POAG.

**SNP**	**OR (95% CI)**	**p**
C1328G>C	1.06 (0.07–17.03)	0.97
C1394T>C	0.35 (0.01–8.69)	0.53
C1557G>C	0.66 (0.03–16.39)	0.80
C1572T>C	0.40 (0.02–8.31)	0.55
C1925T>C	1.29 (0.68–2.47)	0.43
C2016C>G	0.18 (0.01–3.23)	0.24
C503G>A	0.35 (0.01–8.69)	0.53
C685G>A	1.34 (0.52–3.42)	0.55
rs10175368	0.94 (0.81–1.09)	0.42
rs10916	1.13 (0.96–1.33)	0.14
rs162549	0.96 (0.82–1.13)	0.64
rs162556	1.13 (0.99–1.29)	0.07
rs162562	1.14 (0.97–1.34)	0.11
rs2617266	0.94 (0.53–1.68)	0.84
rs180040	1.18 (0.60–2.32)	0.63
rs1056836	0.67 (0.37–1.22)	0.19
rs10012	1.00 (0.78–1.28)	0.99
rs1056827	1.18 (0.83–1.70)	0.36
rs1056837	0.83 (0.60–1.16)	0.28
rs2567206	1.12 (0.74–1.69)	0.60

Based on our meta-analyses and results reported in individual studies.

### Gene-based analysis

Using the p-values obtained from association of individual SNPs with POAG, we performed a gene-based association study to examine the cumulative effect of these genetic polymorphisms on POAG. None of the four methods indicated an association of *CYP1B1* with POAG (all p>0.5, Table 4).

**Table 4:** Gene-based analysis of genetic polymorphisms in *CYP1B1* with POAG.

**Gene**	**Fisher**	**Simes**	**Inverse**	**TPM**
*CYP1B1*	0.63	0.80	0.54	0.73

## Discussion

In this paper, we conducted a systematic search and conducted meta- and gene-based analyses of the genetic polymorphisms in *CYP1B1* with POAG. Our meta-analyses did not detect any statistically significant associations between polymorphisms of *CYPIB1* and POAG. The gene-based analysis indicated that there is no significant association of PAOG with the cumulative effects of the genetic polymorphisms in *CYP1B1*. To the best of our knowledge, this is the first study on the association of polymorphisms in *CYP1B1* with POAG through a meta- and gene-based approach.

Genetic factors play a critical role in predisposition to POAG. However, the genetic pathophysiology of glaucoma remains largely unknown, with mutations in known genes accounting for less than 15% of the disease [29]. Two genes (*MYOC* and *OPTN*) have been reported to show a causative relationship with POAG [5,7]. Mutations of myocilin protein could obstruct the outflow of the aqueous humor through the trabecular meshwork, leading to a markedly elevated IOP. Mice with myocilin mutations in Tyr437His have shown POAG symptoms, such as elevated IOP, RGC death, and axonal degeneration in the optic nerve [36-38]. Optineurin protein might play a role in the protection of the optic nerve from tumor necrosis factor-mediated apoptosis, and mutations in optineurin could lead to loss of function of this protein, which could decrease the threshold for ganglion cell apoptosis in patients with glaucoma [7].

Mutations in *CYP1B1* also have been reported to be implicated in POAG. *CYP1B1* (OMIM 601771) is located on chromosome 2p21 at the GLC3A locus. Being a member of the cytochrome p450 gene superfamily of monooxygenase, it recently became known for its role in eye development during embryogenesis [39]. Studies have shown that the frequency of *CYP1B1* mutations varies in patients across countries–5.0% in Canada [40], 4.6% in France [41], 10.9% in Spain [21], 4.5% in Eastern India [30], and 10.8% in Southern India [42]. Previous studies have indicated a minor involvement of the mutations in *CYP1B1* in the pathogenesis of juvenile open-angle glaucoma (JOAG) and POAG [20,24,26,28,29]. In addition, *CYP1B1* mutations have been suggested to be associated with clinical features like optic disc cupping and visual field loss [34]. One study showed that *CYP1B1*^−/−^ mice exhibited structural abnormalities in the ocular drainage structures, similar to human primary congenital glaucoma (PCG) [43,44]. However, the exact mechanism by which *CYP1B1* contributes to glaucoma remains unknown.

There are a few studies on the effects of genetic polymorphisms in *CYP1B1* on POAG, with conflicting results being reported [22,30-34]. One study revealed a significant association of a common SNP, L432V (rs1056836), in *CYP1B1* with POAG in the Indian population (OR=6.03, 95% CI 3.86–9.40; p<0.001) [31]. Other studies failed to detect a significant association [30,32-34]. Similarly, the study by Melki et al. [34] reported a significant association of another common SNP, N453S (rs180040), with POAG in French patients (OR=3.48, 95% CI=2.34–5.15; p<0.001), but no significant association was found in other studies [30-33]. It is unclear what factors contribute to the conflicting results reported in these studies. We found significant heterogeneity for the meta-analyses of the two SNPs (both p<0.001) and speculate that different genetic structures among the different populations might be an important factor accounting for the disparate results. For example, the minor allele (G) frequency of rs180040 in Caucasians is about 19% (Hapmap database), while in the Chinese population it is reported to be 0.5% in POAG patients and 0 in controls [33] (0.4% in Hapmap database). Of course, other factors, such as environment and diet, might play roles in these differences as well.

There are certain limitations to this study. First, due to the limited availability of published results, the number of studies included in each meta-analysis is relatively small. We could only perform meta-analysis for six SNPs in *CYP1B1*. The association of the remaining 14 SNPs was based on the results from single studies. We expect that as more studies become available, a more accurate estimation of the relationship of *CYP1B1* with POAG will be obtained. Second, definition of POAG was not consistent across the six studies for the meta- and gene-based analyses (Appendix 1). Third, although efforts have been made, there are some studies that used different genetic models. One of our assumptions for the meta- and gene-based analyses is that different genetic models should provide similar associations, which might not hold in reality.

In summary, we conducted a meta- and gene-based analysis of the association of POAG with genetic polymorphisms in *CYP1B1*. We did not detect any SNP showing significant association with POAG, and the gene-based analysis indicated that, based on current evidence from published studies, the cumulative effect of polymorphisms in *CYP1B1* is not significantly associated with POAG.

## Appendix 1. Definition of POAG used by the six studies.

POAG: primary open-angle glaucoma; NTG: normal-tension glaucoma; JOAG: juvenile open-angle glaucoma. To access the data, click or select the words “Appendix 1.” This will initiate the download of a compressed (pdf) archive that contains the file.
